# Insertion of an intrathecal catheter in parturients reduces the risk of post-dural puncture headache: A retrospective study and meta-analysis

**DOI:** 10.1371/journal.pone.0180504

**Published:** 2017-07-05

**Authors:** Jiali Deng, Lizhong Wang, Yinfa Zhang, Xiangyang Chang, Xingjie Ma

**Affiliations:** 1 Department of Anesthesia, Jiaxing Maternity and Child Health Hospital, School of Medicine, Jiaxing University, Jiaxing, Zhejiang, China; 2 Department of Cardiothoracic Surgery, First Hospital of Jiaxing, School of Medicine, Jiaxing University, Jiaxing, Zhejiang, China; Massachusetts General Hospital, UNITED STATES

## Abstract

This study aimed to determine whether insertion of an intrathecal catheter following accidental dural puncture (ADP) in obstetric patients can reduce the incidence of post-dural puncture headache (PDPH) and the requirement of a therapeutic epidural blood patch (TEBP). This was also compared with relocating the epidural catheter at a different vertebral interspace. A retrospective study was performed, as well as a meta-analysis of the literature to further validate our findings. We reviewed the records of 86 obstetric patients who suffered from ADP during epidural anesthesia or combined spinal-epidural anesthesia from October 2015 to November 2016 at our institution. Although, there was no significant decrease in the incidence of PDPH (*P* = 0.08), the requirement for a TEBP (*P* = 0.025) was significantly reduced in the intrathecal catheter group compared with the relocated group. In the meta-analysis, 13 eligible studies including 1044 obstetric patients were finally identified. To estimate the pooled risk ratios (RRs), fixed or random effect models were used depending on the heterogeneity. We initially found that an intrathecal catheter significantly reduced the incidence of PDPH (pooled RR = 0.823; 95% CI = 0.700–0.967; *P* = 0.018) and the requirement of a TEBP (pooled RR = 0.616; 95% CI = 0.443–0.855; *P* = 0.004). Our study shows that insertion of an intrathecal catheter following ADP might be an effective and dependable method for reducing the risk of a PDPH and requirement for a TEBP in obstetric patients.

## Introduction

Neuraxial analgesia is the most effective and reliable technique for pain relief during labor, and is widely used in maternity units [1]. Accidental dural puncture (ADP) is one of the most common complications of neuraxial anesthesia in labor, with an incidence between 0.19% and 3.6% [2]. Subsequent post-dural puncture headache (PDPH) develops in more than 50% of patients [3]. The clinical symptomatology of PDPH can destroy the joy of childbirth, hinder nursing the infants, prolong hospital stay, and increase costs, resulting in dissatisfaction of the experience of anesthesia.

Nevertheless, there are no accepted guidelines for immediate management of ADP. Based on the clinical experience and preference of anesthesiologists, they can choose to relocate the epidural catheter at a different vertebral interspace or insert an intrathecal catheter to achieve analgesia during labor. Traditionally, relocating the epidural catheter was widely applied. However, in recent years, insertion of an intrathecal catheter following APD has gained popularity at our institution. Nevertheless, the efficacy of an intrathecal catheter in reducing the risk of PDPH and requirement for a TEBP remains controversial.

This study aimed to assess whether an intrathecal catheter could decrease the incidence of PDPH and the requirement of a TEBP compared with relocating the catheter. We conducted a retrospective study and systematically reviewed all related articles in an attempt to provide a more objective assessment of intrathecal catheters.

## Materials and methods

### Patients

This was a single-center, retrospective study that was conducted in the Jiaxing Maternity and Child Health Hospital. This hospital is the biggest obstetrics and gynecology hospital in Jiaxing city with a population of more than 5 million. We retrospectively analyzed anesthetic records of 15,632 obstetric patients who underwent epidural anesthesia or combined spinal-epidural anesthesia for labor pain relief since October 2015 to November 2016. Obstetric patients who had a past medical history of headaches, preeclampsia, or eclampsia were excluded. Finally, a total of 86 obstetric patients who suffered from ADP were identified in our study. Among them, 47 immediately received an intrathecal catheter following ADP at the first attempt. The other 39 patients were managed by relocating the epidural catheter at a different vertebral interspace. The incidence of PDPH and the requirement for a TEBP between these two management protocols were compared.

The Jiaxing Maternity and Child Health Hospital Institutional Review Board approved the study protocol with waiver of informed consent. All personal information was de-identified and medical records were analyzed anonymously to protect patient privacy.

### Literature search and selection criteria

A literature search was performed via PubMed, EMbase, and Web of Science databases for relevant articles. Search items of “accidental dural puncture” OR “inadvertent dural puncture” OR “unintentional dural puncture” and “intrathecal catheter” were used in the literature search. The last search was performed on January 31, 2017. We also manually screened the reference lists of relative articles to prevent missing any studies. We contacted the authors of eligible studies if the original articles failed to present sufficient information. A comprehensive literature search was conducted independently by Xingjie Ma and Jiali Deng. All disagreements were resolved by discussion between the two researchers. Eligible studies were selected on the basis of the following criteria: (1) the population was defined as obstetric patients who received epidural anesthesia or combined spinal-epidural anesthesia who had ADP, (2) the immediate measure included insertion of an intrathecal catheter, (3) the incidence of PDPH was reported in a dichotomous form, and (4) reviews, letters, conference abstracts and other non-original articles were excluded. The PRISMA statement could be found in S1 Table.

### Data extraction

The following data were extracted from all eligible studies: first author’s name, published year, country, number of patients, delivery mode, study type, and outcomes. Data extraction was independently conducted by two investigators with the use of manufactured forms. If data or methodological details were absent, the first author was contacted. If after two attempts at contact no reply was received, the trials were only included if sufficient information was available. For studies with more than one study group, only relevant groups for this review were included.

### Statistical analysis

The incidence of PDPH and the requirement for a TEBP were reported as risk ratios (RRs) with 95% confidence intervals (CIs), and were analyzed in our study. Statistical analysis was performed using the Student’s *t* test, chi-square test, and Fisher’s exact test as appropriate. Heterogeneity was assessed using Q and I^2^ statistics [4, 5]. If the P value was less than 0.10 and I^2^ exceeded 50%, indicating the presence of heterogeneity, a random effects model was used. Otherwise, the fixed effect model was adopted. Publication bias was assessed via visual inspection of Begg’s funnel plots [6]. Publication bias was formally tested using Egger’s regression asymmetry method with results considered to indicate potential bias when *P*<0.10[7]. All statistics were performed using SPSS software version 20 (SPSS, Chicago, IL, USA) and STATA version 12.0 (STATA Corporation, College Station, TX, USA). Significance was set at *P*<0.05 for all analysis.

## Results

We defined ADP as cases in which cerebrospinal fluid flowed through an 18 gauge Tuophy needle during puncture. A total of 89 obstetric patients who suffered from ADP after epidural or combined spinal-epidural blocks were reviewed. Among them, three patients were excluded because of a history of head injury or medical headache. Finally, we included 86 patients in this retrospective study, in which 47 patients had an intrathecal catheter inserted through a dural puncture hole (ITC group). A total of 39 patients had epidural catheters relocated (Relocated group). The demographic characteristics of the patients were compared between the two groups (Table 1). Although there was no significant decrease in the incidence of PDPH (*P* = 0.08), the requirement for a TEBP (*P* = 0.025) was significantly reduced in the ITC group compared with the relocated group (Table 2).

**Table 1:** Comparison of demographic characteristics between both groups (ITC group versus relocated group).

	ITC group (n = 47)	Relocated group (n = 39)	*P* value
Age	30.7±3.7	29.9±3.2	0.479
weight	72.2±9.7	73.2±10.4	0.761
height	160.5±5.0	159.4±4.6	0.444
BMI	28.0±3.3	28.8±3.5	0.464
Parity			0.591
Primiparous	22(46.8%)	16(41.0%)	
Multiparous	25(53.2%)	23(59.0%)	
Delivery mode			0.738
Vaginal	27(57.4%)	21(53.8%)	
Caesarean	20(42.6%)	18(46.2%)	

BMI: body mass index; Data are mean ± SD or number (%).

**Table 2:** The incidence of PDPH and the requirement for a TEBP in the ITC group versus the relocated group.

	ITC group (n = 47)	Relocated group (n = 39)	*P* value
PDPH	20(42.6%)	24(61.5%)	0.08
TEBP	13(27.7%)	20(51.3%)	0.025

PDPH: post dural puncture headache; TEBP: therapeutic epidural blood patch; Data are mean ± number (%).

**Fig 1:**
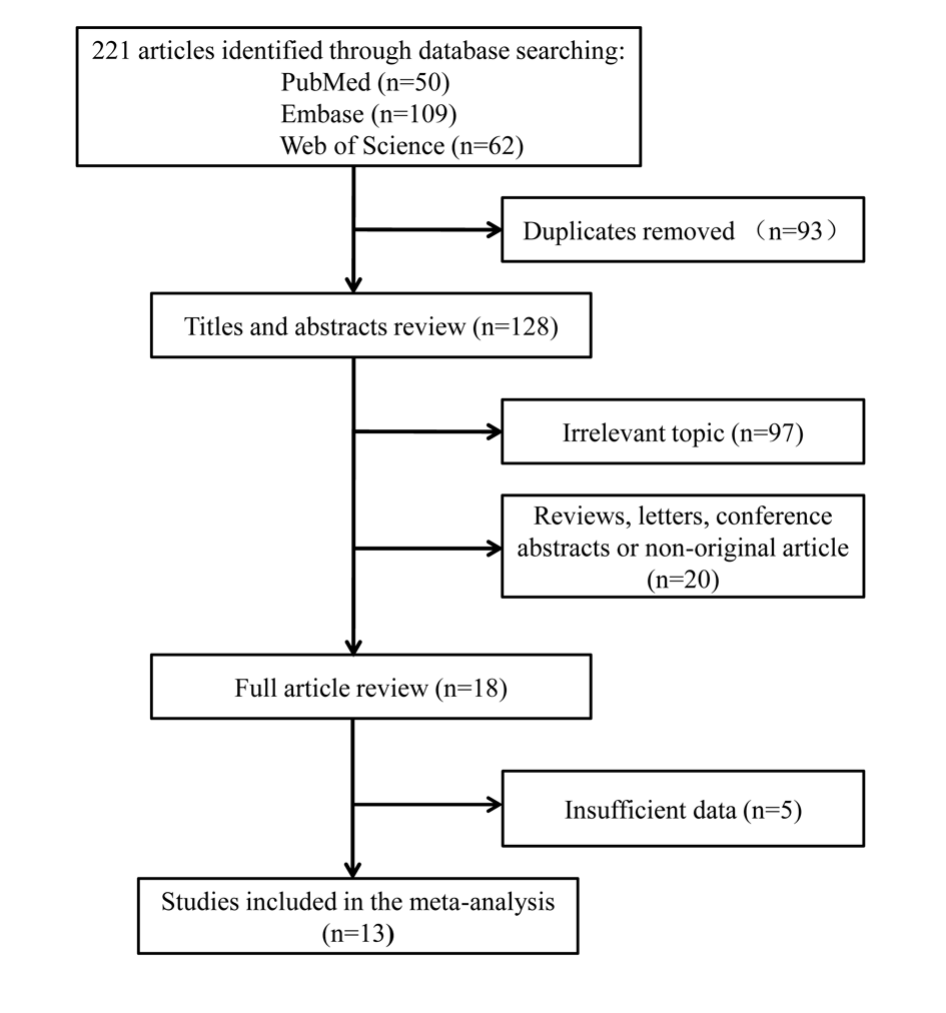
Flow diagram of the selection process and specific reasons for exclusion in the meta-analysis.

**Fig 2:**
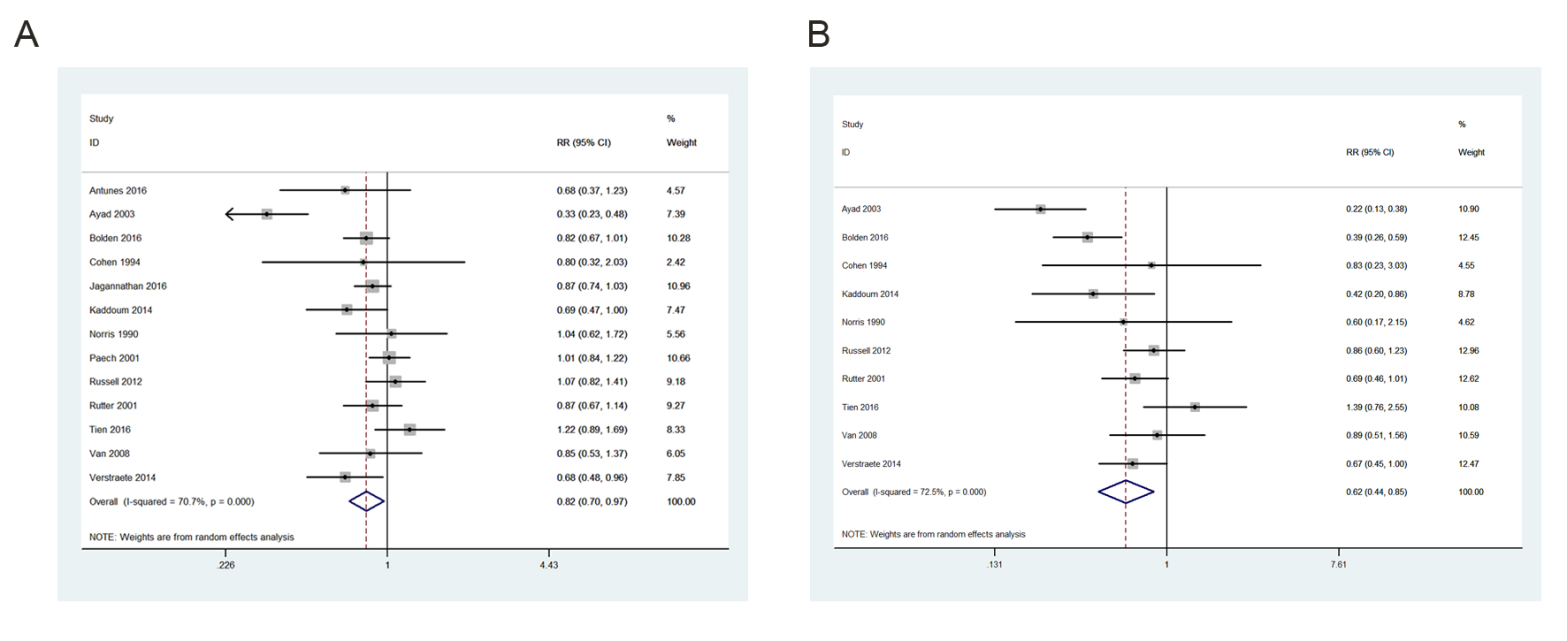
Forest plot of RRs for PDPH (A) and a TEBP (B) in obstetric patients.

**Fig 3:**
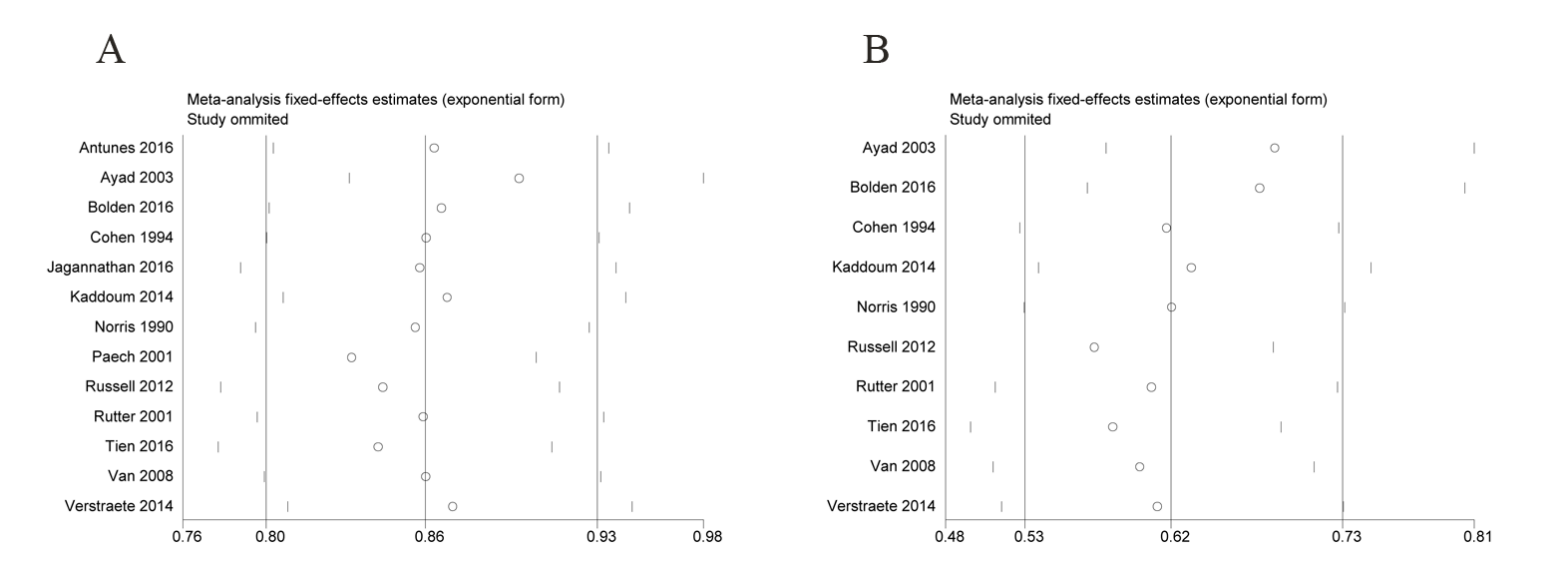
Sensitivity analysis on the effect of each individual study on the overall meta-analysis of PDPH (A) and a TEBP (B).

**Fig 4:**
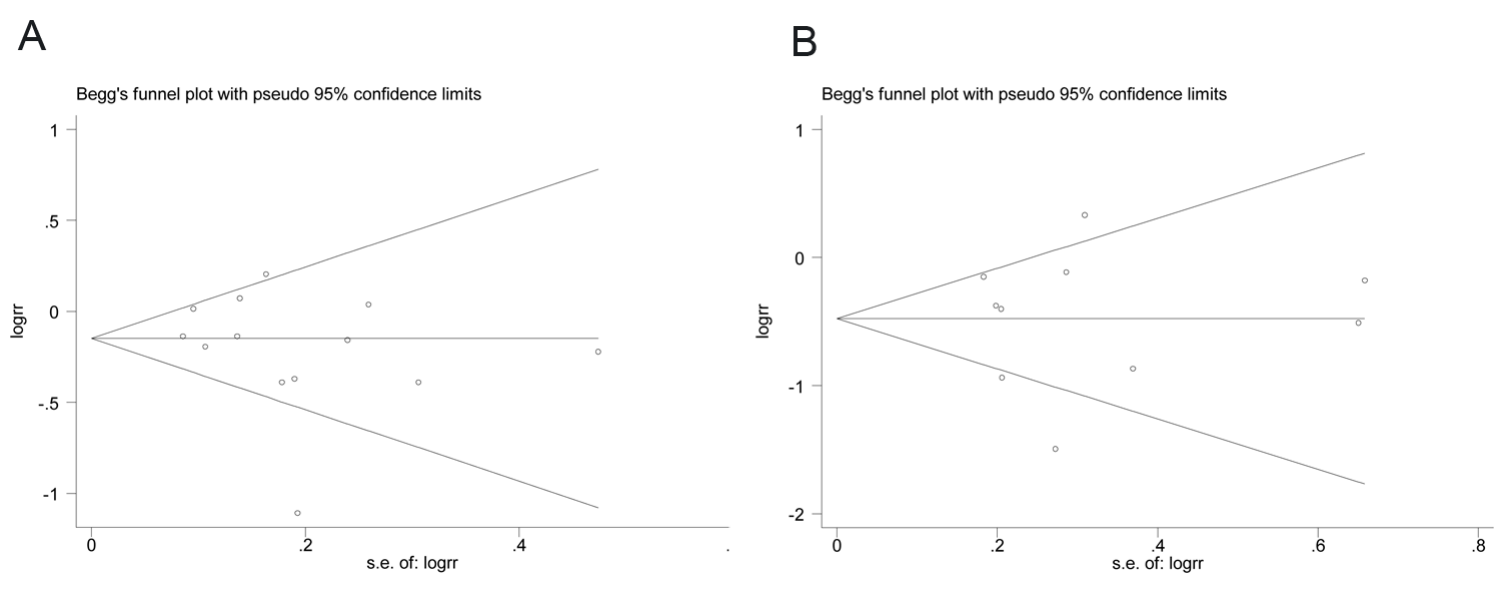
Begg’s funnel plots of studies that examined PDPH (A) and a TEBP (B) as a test for publication bias.

In our systematic literature review, 221 articles were initially identified and 13 studies including 1044 obstetric patients were finally included in the meta-analysis [1, 3, 8–18]. Fig 1 shows the selection process for eligible studies. The main characteristics of the included studies are shown in Table 3. Our meta-analysis showed a significant reduction in PDPH (pooled RR = 0.823; 95% CI = 0.700–0.967; P = 0.018) and requirement for a TEBP (pooled RR = 0.616; 95% CI = 0.443–0.855; P = 0.004) in the ITC group compared with relocated group (Fig 2). The Q test showed significant heterogeneity. We further performed sensitivity analysis to investigate whether the studies were convincing and stable. We found no significant change in pooled RRs when any of the enrolled studies were excluded (Fig 3). Therefore, we concluded that no individual study dominated the meta-analysis results and the credibility of the outcomes was validated. Publication bias was evaluated by Begg’s funnel plots and Egger’s test. Begg’s funnel plots did not show any evidence of significant asymmetry in the meta-analysis of PDPH (*P* = 0.300) and a TEBP (*P* = 0.721) (Fig 4). Egger’s test also showed the absence of publication bias in PDPH (*P* = 0.335) and a TEBP (*P* = 0.954).

**Table 3:** Characteristics of 13 enrolled studies included in the meta-analysis.

First author (year)	Country	Number of patients	Delivery mode	Study Type	Outcomes
Paech (2001)	Australia	75	Vaginal	Prospective study	PDPH
Antunes (2016)	Portugal	54	Vaginal/caesarean	Retrospective study	PDPH
Ayad (2003)	USA	103	Vaginal	Retrospective study	PDPH, TEBP
Bolden (2016)	USA	218	Vaginal/caesarean	Retrospective study	PDPH, TEBP
Cohen (1994)	USA	45	caesarean	Retrospective study	PDPH, TEBP
Jagannathan (2016)	USA	236	Vaginal/caesarean	Retrospective study	PDPH
Kaddoum (2014)	Lebanon	238	Vaginal	Retrospective study	PDPH, TEBP
Russell (2012)	UK	115	Vaginal/caesarean	Prospective study	PDPH, TEBP
Rutter (2001)	UK	71	Vaginal	Retrospective study	PDPH, TEBP
Tien (2016)	USA	109	Vaginal/caesarean	Retrospective study	PDPH, TEBP
Van (2008)	Belgium	55	Vaginal/caesarean	Retrospective study	PDPH, TEBP
Verstraete (2014)	UK	128	Vaginal/caesarean	Retrospective study	PDPH, TEBP
Norris (1990)	USA	56	Vaginal	Retrospective study	PDPH, TEBP

PDPH: post dural puncture headache; TEBP: therapeutic epidural blood patch

## Discussion

Our findings add to the growing body of evidence that insertion of an intrathecal catheter following ADP is an effective measure to reduce the risk of PDPH and requirement for a TEBP in obstetric patients. In our retrospective study, we found that although there appeared to be a reduction in the incidence of PDPH in the ITC group, this was not significant (*P* = 0.08). However, the requirement of a TEBP was significantly lower in the ITC group than in the relocated group (*P* = 0.025). Furthermore, our meta-analysis showed that insertion of an intrathecal catheter significantly reduced the incidence of PDPH (pooled RR = 0.823; 95% CI = 0.700–0.967; P = 0.018) and the requirement for a TEBP (pooled RR = 0.616; 95% CI = 0.443–0.855; P = 0.004).

The difference between our retrospective study and the meta-analysis may have been due to multicenter studies and a larger sample size of the meta-analysis (our retrospective study only included 86 patients compared with 1044 patients in the meta-analysis). A small difference cannot be observed by a small sample size. The difference in incidence of PDPH is small between the groups. Several studies that only included a small size population failed to detect a difference in the incidence of PDPH [12, 15, 18], while our meta-analysis of 13 studies with 1044 patients observed a significant difference. Even when our retrospective study was included in the meta-analysis, we found similar results (PDPH: pooled RR = 0.814, 95% CI = 0.698–0.950, *P* = 0.009; TEBP: pooled RR = 0.608, 95% CI = 0.451–0.819, *P* = 0.001).

A previous meta-analysis performed by Apfel et al [19] reported that insertion of an intrathecal catheter did not decrease the risk of PDPH and the requirement for a TEBP, but this meta-analysis only included three studies. A later meta-analysis by Hessen et al [20] concluded that insertion of an intrathecal catheter significantly reduced the requirement for a TEBP (pooled RR = 0.64, 95% CI = 0.49–0.84, *P* = 0.001), but did not decrease the incidence of PDPH (pooled RR = 0.82, 95% CI = 0.67–1.01, P = 0.06). Although this meta-analysis included nine reports, among them, three were abstracts of a conference, and they were not published at full length. Since the last meta-analysis was performed, several relevant studies have been published [1, 3, 8, 13, 14, 18]. Most interestingly, Verstraete et al [8] and Kaddoum et al [13] both reported that the application of intrathecal catheter following ADP reduced the risk of PDPH in obstetric patients. In our meta-analysis, we included 13 eligible studies published as full articles. We performed a more comprehensive assessment on this debate and included a larger sample size compared with other meta-analyses.

Some limitations of our study should be pointed out. First, our retrospective study was a single-center study, the sample size and available resources were limited. We only investigated whether insertion of an intrathecal catheter following ADP in obstetric patients could reduce the incidence of PDPH and the requirement of a TEBP compared with relocating an epidural catheter at a different vertebral interspace. Secondly, a major limitation of our meta-analysis is the relatively low level of quality of the included studies as demonstrated by statistical heterogeneity. This affected the accuracy of our quantitative and qualitative assessments. Although we did not observe publication bias in our meta-analysis, it could still have been present because the published results might not be representative of the conducted studies. The presence of high heterogeneity among the included studies was obvious. Therefore, we performed our meta-analysis with the random effect model. Furthermore, sensitivity analysis showed that the high level of heterogeneity did not affect the summary estimate. Finally, among 13 included studies in our meta-analysis, the study by Paech et al [16] is the only randomized study.

In conclusion, our study indicates that insertion of an intrathecal catheter following ADP might be an effective method for reducing the risk of PDPH and the requirement for a TEBP in obstetric patients. Large, prospective, multicenter studies are required to confirm our findings.

## Supporting information

S1 Table PRISMA checklist. (DOC)
